# PI3K isoform inhibition associated with anti Bcr-Abl drugs shows in vitro increased anti-leukemic activity in Philadelphia chromosome-positive B-acute lymphoblastic leukemia cell lines

**DOI:** 10.18632/oncotarget.15542

**Published:** 2017-02-20

**Authors:** Simona Ultimo, Carolina Simioni, Alberto M. Martelli, Giorgio Zauli, Camilla Evangelisti, Claudio Celeghini, James A. McCubrey, Giorgia Marisi, Paola Ulivi, Silvano Capitani, Luca M. Neri

**Affiliations:** ^1^ Department of Morphology, Surgery and Experimental Medicine, University of Ferrara, Ferrara, Italy; ^2^ Department of Biomedical and Neuromotor Sciences, University of Bologna, Bologna, Italy; ^3^ Institute of Molecular Genetics, Rizzoli Orthopedic Institute, National Research Council, Bologna, Italy; ^4^ Department of Life Sciences, University of Trieste, Trieste, Italy; ^5^ Department of Microbiology and Immunology, Brody School of Medicine, East Carolina University, Greenville, NC, USA; ^6^ Biosciences Laboratory, Istituto Scientifico Romagnolo per lo Studio e Cura dei Tumori (IRST) IRCCS, Meldola, Italy; ^7^ LTTA Center, University of Ferrara, Ferrara, Italy

**Keywords:** PI3K isoforms, Bcr-Abl, Ph^+^ B-acute lymphoblastic leukemia, autophagy, tyrosine kinase inhibitors

## Abstract

B-acute lymphoblastic leukemia (B-ALL) is a malignant disorder characterized by the abnormal proliferation of B-cell progenitors. Philadelphia chromosome-positive (Ph^+^) B-ALL is a subtype that expresses the Bcr-Abl fusion protein which represents a negative prognostic factor. Constitutive activation of the phosphatidylinositol 3-kinase/Akt/mammalian target of rapamycin (PI3K/Akt/mTOR) network is a common feature of B-ALL, influencing cell growth and survival. In the present study, we aimed to investigate the efficacy of PI3K isoform inhibition in B-ALL cell lines harboring the Bcr-Abl fusion protein.

We studied the effects of anti Bcr-Abl drugs Imatinib, Nilotinib and GZD824 associated with PI3K isoform inhibitors. We used a panel of six compounds which specifically target PI3K isoforms including the pan-PI3K inhibitor ZSTK474, p110α BYL719 inhibitor and the dual p110γ/p110δ inhibitor IPI145. The effects of single drugs and of several drug combinations were analyzed to assess cytotoxicity by MTS assays, apoptosis and autophagy by flow cytometry and Western blot, as well as the phosphorylation status of the pathway.

ZSTK474, BYL719 and IPI145 administered in combination with imatinib, nilotinib and GZD824 for 48 h, decreased cell viability, induced apoptosis and autophagy in a marked synergistic manner.

These findings suggest that selected PI3K isoform inhibitors used in combination with anti Bcr-Abl drugs may be an attractive novel therapeutic intervention in Ph^+^ B-ALL.

## INTRODUCTION

The over-production of immature white blood cells is a relevant feature of ALL, a heterogeneous disease characterized by multiple, prognostically relevant genetic aberrations.

The Philadelphia (Ph) chromosome harboring the t(9;22) (q34;q11) translocation and the ensuing fusion gene Bcr-Abl lead to an aberrant cell proliferation [1]. Bcr-Abl is the most common cytogenetic abnormality and the most unfavourable prognostic factor in adult acute lymphoblastic leukemia patients (ALL) [2], where 20–30% of them express the Bcr-Abl oncogene [3, 4].

The selective Bcr-Abl tyrosine kinase inhibitor Imatinib deeply changed the pharmacological treatment of another form of Ph^+^ cancer, i.e. chronic myeloid leukemia (CML), by inducing long lasting remissions [5]. Unfortunately, Imatinib showed much less efficiency in treating Ph^+^ ALL and although unrelated to the Bcr-Abl kinase domain alterations, the underlying mechanisms are largely unknown [1]. The second generation tyrosine kinase inhibitor (TKI) Nilotinib shows both a stronger potency than Imatinib and also acts against most Imatinib-unresponsive Bcr-Abl mutation variants [6–8]. A new, third generation orally bioavailable Bcr-Abl inhibitor, GZD824, has been recently developed, with potency against a wide range of Bcr-Abl mutants [9]. In general, Ph^+^ B-ALL is less sensitive to TKIs that CML. Therefore, novel drugs are needed to improve response rates and to circumvent TKI-resistance in Ph^+^ B-ALL [10].

Class I phosphatidylinositol 3-kinases (PI3Ks) comprises members of a conserved family of heterodimeric intracellular lipid kinases capable to activate Akt which in turn phosphorylates target proteins thus affecting cell growth, cell cycle progression, and cell survival [11, 12].

Class IA PI3Ks comprises a catalytic subunit (p110α, p110β, p110γ, p110δ) and a tightly bound regulatory subunit (p85α, p55α, p50α, p85β, or p55γ). The regulatory subunits maintain the integrity of the catalytic one and direct the heterodimer to membrane-associated signaling complexes [13]. The activation of the PI3K network is regulated by the 3′-phosphate lipid phosphatase PTEN (phosphatase and tensin homolog deleted on chromosome 10) and loss of activity of this tumor suppressor gene induces an increased downstream activation [14–16]. Alterations in PTEN have been reported in several tumors, most notably endometrial, central nervous system, skin and prostate cancers [17].

Due to the crucial role of PI3Ks in regulating cell cycle, metabolism, and survival, the PI3K signaling cascade in human leukemias is one of the most often altered pathways [18, 19] and different compounds targeting members of the PI3K network have been developed and entered clinical trials [20]. Currently several PI3K inhibitors are under development: ZSTK474, a specific pan-PI3K inhibitor [21, 22], displays potent anti-tumor efficacy on various solid tumors [23, 24]. PIK3CA, the gene which encodes for p110α PI3K, is mutated in a variety of tumor types [25–27] and BYL719 is a specific class-IA PI3K inhibitor, which acts by binding the ATP-binding domain of the p110α subunit [28]. BYL719 has recently been reported to have significant activity against tumors carrying mutations in the p110α subunit of PI3K [29, 30].

Pharmacological blockade of both p110γ and p110δ reduced T-ALL proliferation and survival, indicating these isoforms as therapeutic targets for T-ALL treatment [31]. AS605240 has been previously described as an isoform-selective ATP-competitive inhibitor of PI3Kγ [32] while CAL-101 is an oral p110δ inhibitor currently under clinical evaluation in patients with B-cell malignancies [33]. The selective, oral p110δ and p110γ subunits inhibitor, IPI145, is reported to be in phase I study [34]. TGX221, is a p110β-selective inhibitor [35].

Aim of this study is to investigate the effects of a panel of PI3K isoform inhibitors in Ph^+^ B-ALL cell lines by combining their anti-tumor activity with anti Bcr-Abl drugs. We decided to specifically target p110α, p110β, p110γ and p110δ PI3K catalytic subunits, along with dual p110γ/p110δ and pan-PI3K inhibitors, and we evaluated their effects on leukemic cell proliferation and survival. Our results demonstrated that the pan PI3K isoform inhibitor ZSTK474, the p110α inhibitor BYL719 and the IPI145 dual p110γ/p110δ inhibitor exerted the most powerful effects in all the Ph^+^ B-ALL cell lines tested and their combined use with TKIs enhanced the treatment efficacy. Our findings support the concept that clinical trials examining PI3K inhibitors in combination with TKIs are warranted in patients with Ph^+^ B-ALL.

## RESULTS

### PI3K inhibitors affect cell viability of Ph^+^ B-ALL cell lines

**Figure 1:**
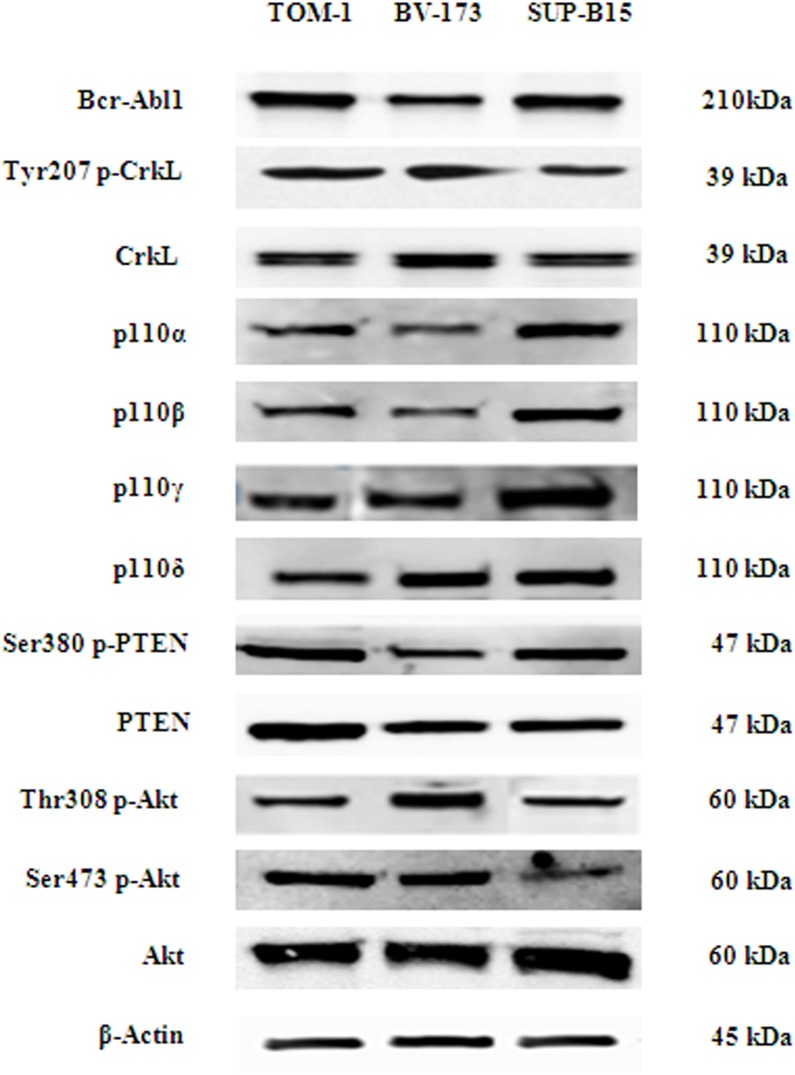
Bcr-Abl protein level, PI3K catalytic subunits expression (p110α, p110β, p110γ, p110δ), and phosphorylation level of PI3K/Akt/mTOR pathway key substrates in Ph^+^ B-ALL cell lines Western blot analysis of Ph^+^ B-ALL cell lines to detect the expression of Bcr-Abl protein, PI3K catalytic subunits and phosphorylation levels of PTEN, CrkL and Akt protein. Twenty-five μg of protein were blotted on each lane. β-actin was revealed as loading control. One representative of three different blots is shown.

In order to establish the role of the different PI3K catalytic subunits in sustaining leukemic cells proliferation and survival, we investigated by Western blot analysis the baseline expression of Bcr-Abl and its substrate CrkL, the PI3K catalytic subunits and the key enzymes of the PI3K/Akt/mTOR network in three B-ALL cell lines (TOM-1, BV-173 and SUP-B15). As shown in Figure 1, Bcr-Abl and CrkL, the p110α, -β, -γ, -δ, PI3K catalytic subunits and the downstream PI3K substrate Akt and the pathway negative regulator PTEN were expressed and/or phosphorylated in all cell lines.

**Figure 2:**
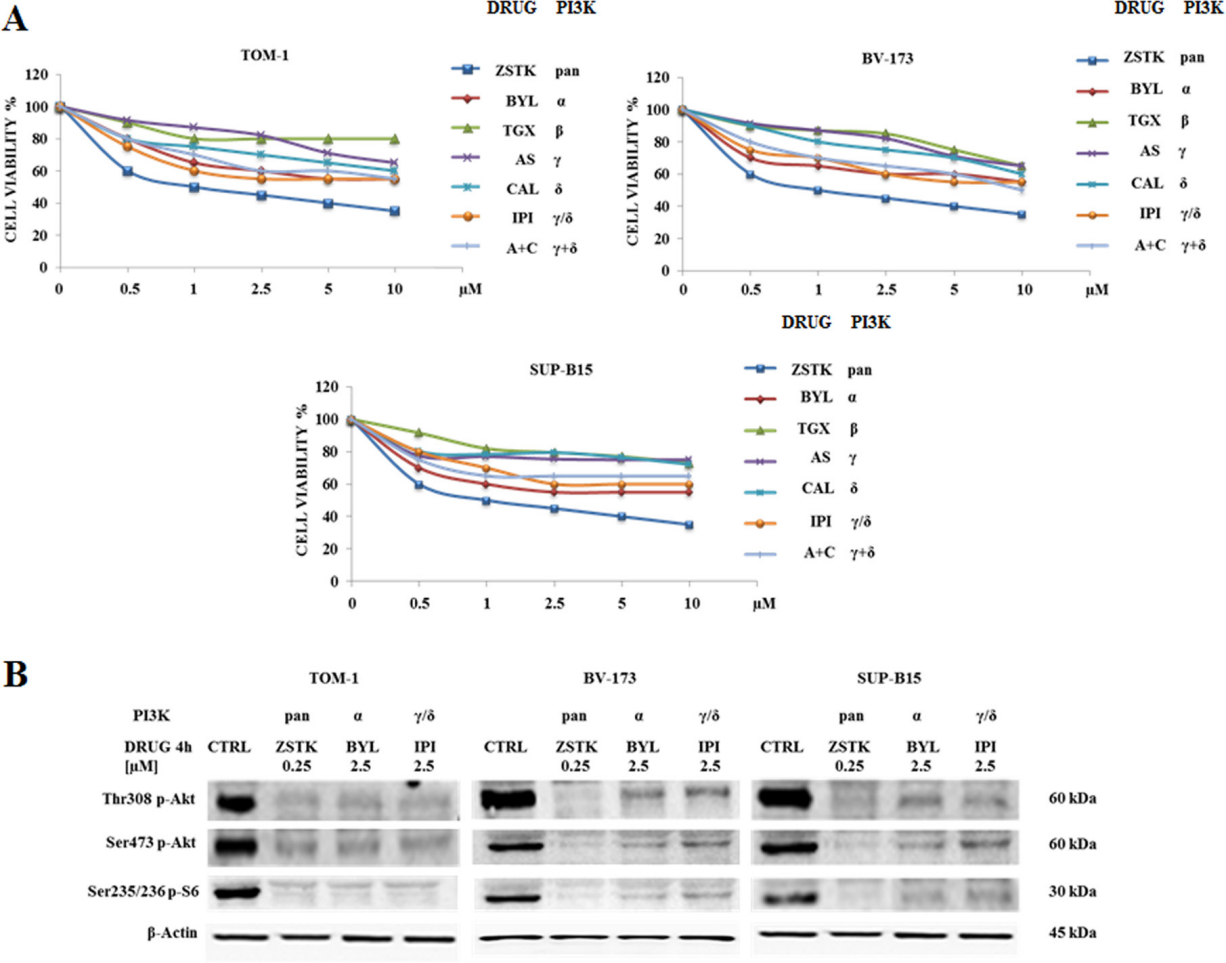
Cytotoxicity of PI3K isoform inhibitors and modulation of PI3K/Akt/mTOR pathway in TOM-1, BV-173 and SUP-B15 cell lines (**A**) MTS assays of Ph^+^ B-ALL cell lines treated for 48 h with increasing concentrations of ZSTK474, BYL719, TGX221, AS605240, CAL101, IPI145 and AS605240 combined with CAL101. ZSTK474, BYL719, TGX221, AS605240, CAL101 and IPI145 inhibitors were shortened in ZSTK, BYL, TGX, AS, CAL, IPI and A+C respectively. When administered together, AS605240 + CAL101 were used at the same concentrations as specified on the X-axis. Results are the mean of three separate experiments. SD was less than 7%. (**B**) Western blot analysis of TOM-1, BV-173 and SUP-B15 cell lines treated for 4 h with selected PI3K isoform inhibitors ZSTK474, BYL719 and IPI145 to detect the phosphorylation level expression of Akt and S6 ribosomal protein. Twenty-five μg of protein were blotted on each lane. β-actin was revealed as loading control. Control (untreated cells), ZSTK474, BYL719 and IPI145 inhibitors were abbreviated in CTRL, ZSTK, BYL and IPI respectively. One representative of three different blots is shown.

For the inhibition of p110α, p110β, p110γ and p110δ, we employed ZSTK474, BYL719, TGX221, CAL101 and AS605240, whose selectivity has been reported [36]. Because of the prominent role of p110δ and p110γ isoforms in white blood cells, effects of the γ/δ dual inhibitor IPI145, as well as of a combination consisting of CAL101 and AS605240, were also evaluated. Cell lines were cultured with increasing concentrations of the drugs for 48 h followed by metabolic activity assessment by MTS assay (Figure 2A). In all cell lines, cell viability decreased after treatment with ZSTK474 with IC_50_ values close to 0.5 μM. Selective inhibition of p110α, p110β, p110γ and p110δ isoforms had less efficacy, and IC_50_ values were not attained up to a 10 μM concentration. Longer time points (72 h) did not further affect the metabolic activity of inhibitor-treated cell lines (data not shown).

We decided to focus our study on ZSTK474 and γ/δ dual inhibitor IPI145 as well as on the p110α inhibitor BYL719, whose role in this type of leukemia has not been thoroughly investigated yet [30, 37, 38].

The PI3K/Akt/mTOR network, its expression and modulation status of key components were assessed to evaluate the efficacy of these inhibitors. Cells were treated with 0.25 μM ZSTK474, 2.5 μM BYL719 or IPI145 and Western blot analysis was performed. These concentrations were chosen as they produced the maximal effects on protein dephosphorylation at the lowest dose. All the PI3K isoform inhibitors decreased the phosphorylation level of the PI3K/Akt/mTOR axis key substrates Akt and S6 ribosomal protein (Figure 2B).

### Autophagy and apoptosis are induced by PI3K inhibitors in B-ALL cell lines

**Figure 3:**
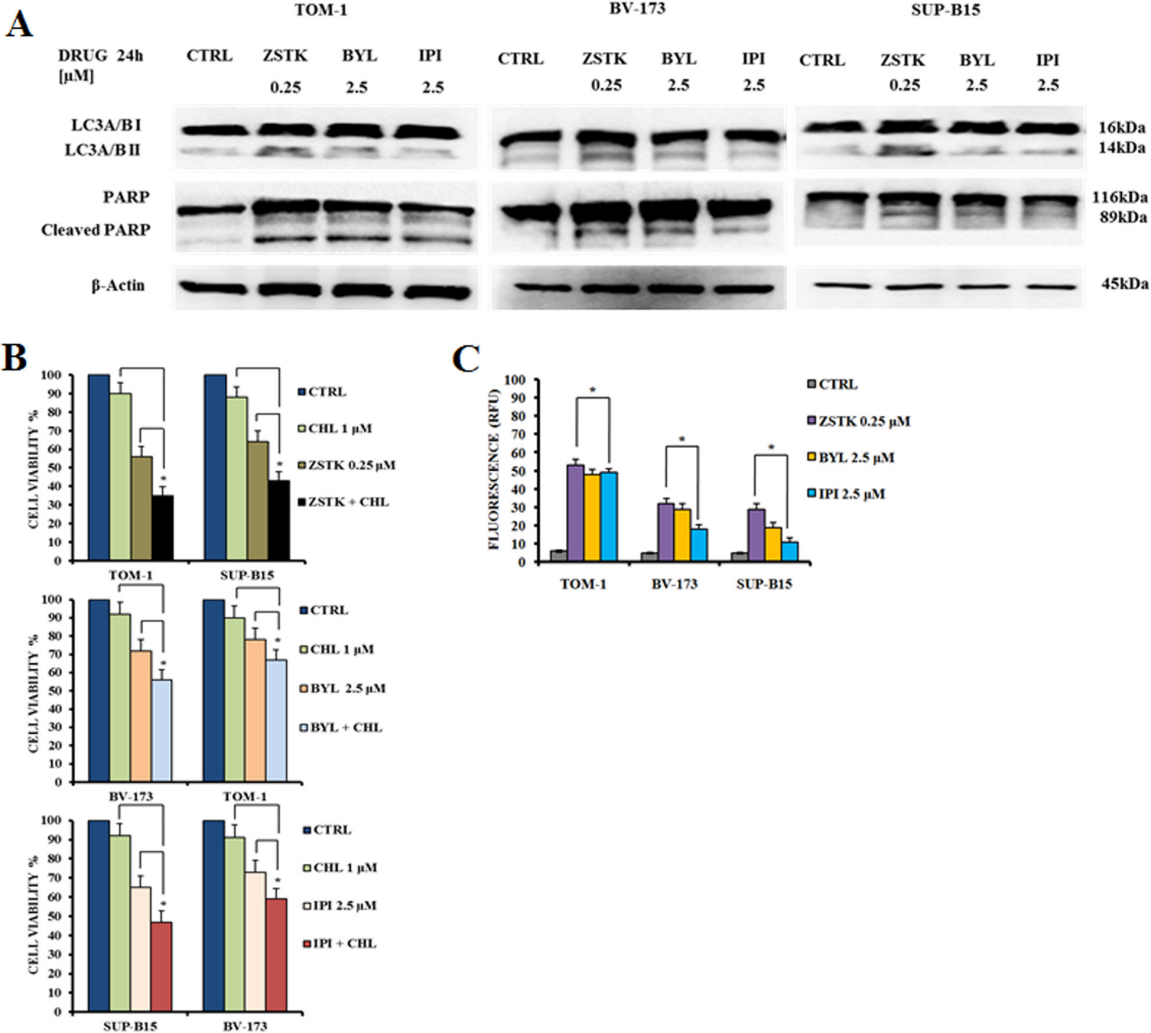
Selected PI3K isoform inhibitors induced autophagy and apoptosis in Ph^+^ B-ALL cell lines (**A**) Western blot analysis documenting the increase of expression of fast-migrating (lipidated) LC3A/B and PARP cleavage in TOM-1, BV-173 and SUP-B15 cells treated for 24 h with ZSTK474, BYL719 and IPI145. Twenty-five μg of protein were blotted on each lane. β-actin documented equal lane loading. Control (untreated cells), ZSTK474, BYL719 and IPI145 inhibitors were abbreviated in CTRL, ZSTK, BYL and IPI respectively. One representative of three different blots is shown. (**B**) MTS assay documenting the effects of the autophagy inhibitor chloroquine (CHL) on the viability of Ph^+^ B-ALL cell lines treated for 24 h with the indicated drugs. Results are the mean of three different experiments ± SD. Asterisks indicate significant differences with respect to untreated cells (*p < 0.05). (**C**) Enzymatic cleavage of the profluorescent substrate Z-DEVD-R110, with release of the intensely fluorescent rhodamine 110-cleaving group, after PI3K inhibitor treatment. The analysis was performed after 24 h of treatment with the drugs. Results are the mean of three different experiments ± SD. Asterisks indicate significant differences with respect to untreated cells (*p < 0.05).

Autophagic process is an evolutionarily mechanism involved in the degradation of cellular components and is crucial for the maintenance of cell homeostasis [39]. Altered autophagy is related with cancer and has been implicated in the disease development and survival [40–42]. To assess whether the administration of PI3K inhibitors could induce autophagy, we analyzed LC3A/B I (non lipidated) and LC3A/B II (lipidated) levels by Western blot in TOM-1, BV-173 and SUP-B15 cell lines after 24 h (Figure 3A). LC3A/B II expression increased with ZSTK474 pan-inhibitor treatment in all cell lines and to a lower extent with BYL719 and IPI145, indicating the induction of autophagy.

To analyze whether the reduced viability was apoptosis-related, cell lines were treated with the inhibitors for 24 h at the same doses used to detect autophagy. Poly (ADP-ribose) polymerase (PARP) cleavage in TOM-1, BV-173 and SUP-B15 cells demonstrated the pro-apoptotic effect induced by the PI3K inhibitors. As demonstrated for the autophagy marker LC3A/B, the expression levels of cleaved PARP increased when cell lines were treated with ZSTK474 pan-inhibitor, or the p110α-selective inhibitor BYL719 and to a lower extent with IPI145 (Figure 3A). The role of autophagy was further investigated using the lysosomal inhibitor chloroquine at 1 μM. In all cases, chloroquine increased the cytotoxic effects of the PI3K inhibitors, suggesting that autophagy is a protective mechanism against these drugs (Figure 3B). Moreover, caspase 3/7 activation was quantified by measuring the enzymatic cleavage of the profluorescent component rhodamine 110, bis-N-CBZ-L-aspartyl-L-glutamyl- L-valyl-L-aspartic acid amide (Z-DEVD-R110), with release of the strongly fluorescent rhodamine 110-cleaving group. The data on caspase 3/7 activity were in agreement with those of Western blot on PARP cleavage (Figure 3C).

### Bcr-Abl inhibitors decreased cell viability in Ph^+^ B-ALL cell lines

**Figure 4:**
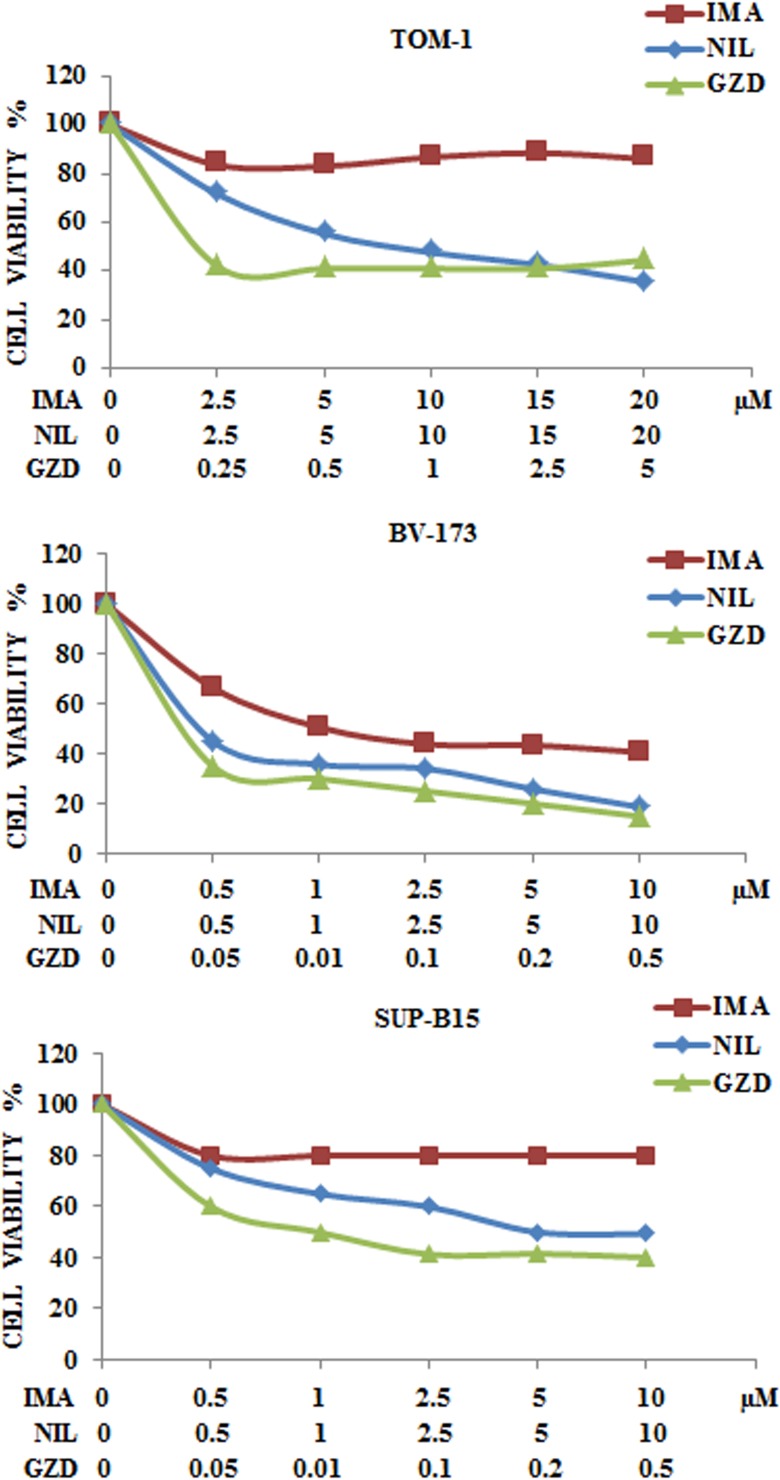
Cytotoxicity of anti Bcr-Abl drugs in TOM-1, BV-173 and SUP-B15 cell lines MTS assays of Ph^+^ B-ALL cell lines treated with increasing concentrations of Imatinib, Nilotinib and GZD824 for 48 h. Results are the mean of three separate experiments. SD was less than 10%. Imatinib, Nilotinib and GZD824 drugs were abbreviated in IMA, NIL and GZD respectively.

We examined by MTS assay the IC_50_ value of each Bcr-Abl inhibitor in the three cell lines. After 48 h of drug administration, the cells showed different drug sensitivity. Imatinib attained an IC_50_ of 1.1 μM in BV-173 cells whereas in TOM-1 and SUP-B15 cell lines IC_50_ was not attained up to a concentration of 20 μM and 10 μM, respectively. Nilotinib IC_50_ ranged from 0.1 μM in BV-173 cells to 7.2 μM in TOM-1 cells. GZD824 showed the greatest potency, with IC_50_ values ranging from 0.03 μM in BV-173 to 0.2 μM in TOM-1 cells (Figure 4 and Table 1).

**Table 1:** IC_50_ and SD values of cells treated for 48 hours with anti Bcr-Abl drugs

	IMATINIB	NILOTINIB	GZD824
TOM-1	>10	7.2 ± 0.57	0.2 ± 0.01
BV-173	1.1 ± 0.07	0.1 ± 0.01	0.03 ± 0.002
SUP-B15	>10	5.8 ± 0.46	0.08 ± 0.005

IC_50_ values are expressed in μM. Results are the mean of three different experiments ± SD.

### Expression levels of pCrkL protein and PI3K/Akt/mTOR signaling pathway substrates

**Figure 5:**
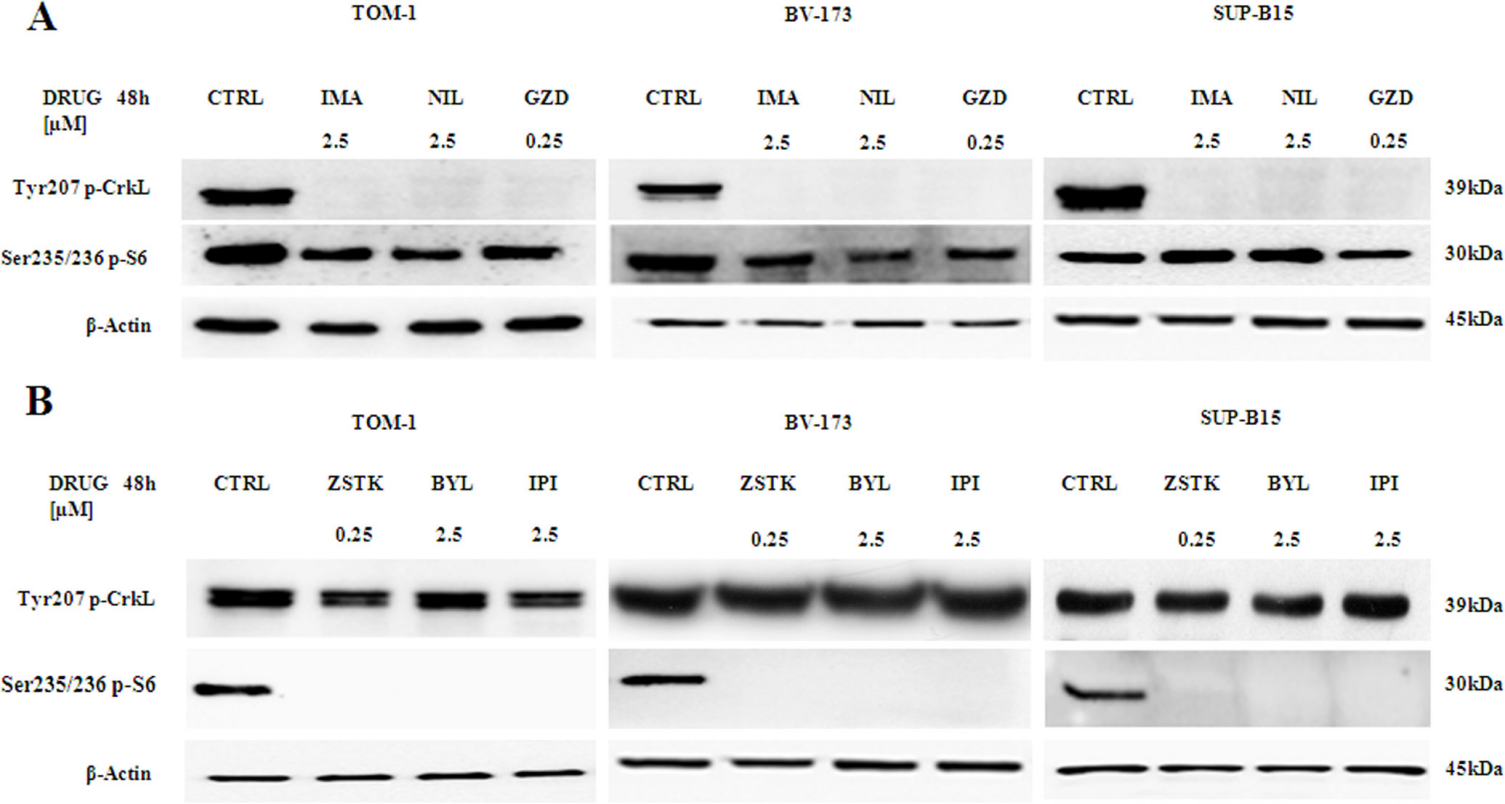
Expression and phosphorylation status of CrkL and S6 ribosomal protein in Ph^+^ B-ALL cell lines (**A**) Western blot analysis of TOM-1, BV-173 and SUP-B15 cells treated for 48 h with 2.5 μM of Imatinib, Nilotinib and 0.25 μM of GZD824. A decrease of the phosphorylated expression of tyrosine kinase CrkL and a slight decrease of the phosphorylated S6 ribosomal protein in all cell lines is shown. Twenty-five μg of protein were blotted on each lane. β-actin documented equal lane loading. One representative of three different blots is shown. Control (untreated cells) was abbreviated in CTRL. Imatinib, Nilotinib and GZD824 drugs were abbreviated in IMA, NIL and GZD respectively. (**B**) Western blot analysis of TOM-1, BV-173 and SUP-B15 cells treated for 48 h with 0.25 μM of ZSTK474 and 2.5 μM of BYL719 and IPI145. Twenty-five μg of protein were blotted on each lane. β-actin documented equal lane loading. One representative of three different blots is shown. Control (untreated cells) was abbreviated in CTRL. ZSTK474, BYL719 and IPI145 inhibitors were abbreviated in ZSTK, BYL and IPI respectively.

Requirement for CrkL kinase activity has been demonstrated in a recent report with Bcr-Abl transformation and oncogenic signal transduction [43]. As shown in Figure 5A, cells were exposed for 48 h to Imatinib, Nilotinib or GZD824, and lysates were analyzed by Western blot with an antibody directed to the phosphorylated forms of CrkL and S6 ribosomal protein. All anti Bcr-Abl drugs decreased the levels of phosphorylated CrkL with little or no effects on S6 ribosomal protein. In contrast, cell treatment with 0.25 μM ZSTK474 or 2.5 μM BYL719 or IPI145 for 48 h did not affect the phosphorylation levels of pCrkL, while the expression of phosphorylated S6 ribosomal protein was abolished (Figure 5B).

### Synergistic cytotoxic effects combining selected PI3K isoform inhibitors with anti Bcr-Abl drugs

**Figure 6:**
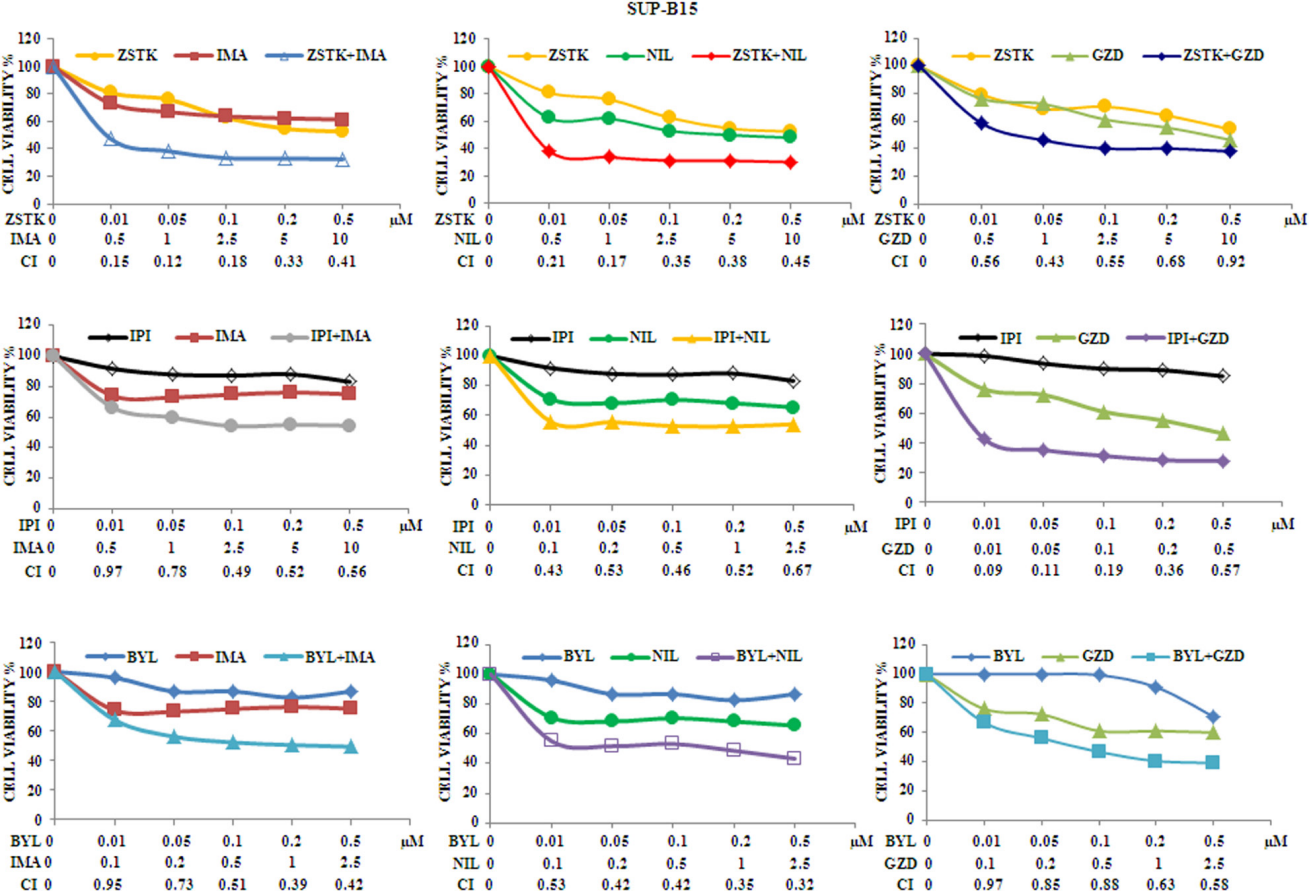
Cytotoxicity and synergism of selected PI3K isoform inhibitors combined with anti Bcr-Abl drugs in SUP-B15 cell line MTS assays of SUP-B15 cells treated with ZSTK474, BYL719, IPI145, Imatinib, Nilotinib and GZD824 alone or with the combinations indicated in the graph legends. The analysis was performed after 48 h of treatment. Results are the mean of three separate experiments. SD was less than 7%. ZSTK474, BYL719, IPI145, Imatinib, Nilotinib and GZD824 inhibitors were abbreviated in ZSTK, BYL, IPI, IMA, NIL and GZD, respectively.

We analyzed whether the combination of ZSTK474, BYL719 or IPI145 with Imatinib, Nilotinib or GZD824 could increase cytotoxic effects on Ph^+^ B-ALL cells. Therefore, MTS assays were performed in SUP-B15 cells treated with the different drugs administered together for 48 h. As shown in Figure 6, SUP-B15 were responsive and showed a good, synergistic cytotoxicity as demonstrated by the CIs.

**Figure 7:**
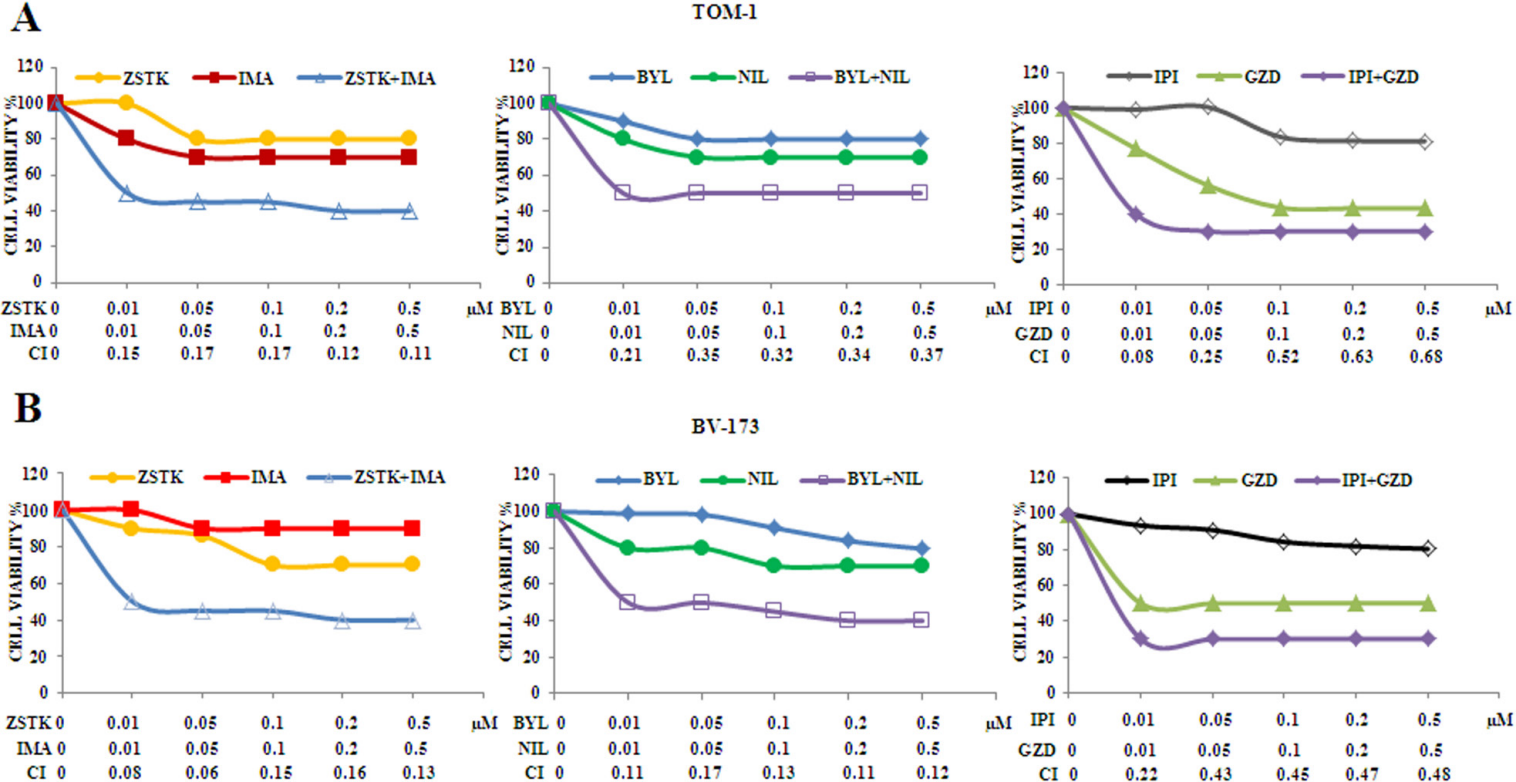
Cytotoxicity and synergism of selected PI3K isoform inhibitors combined with anti Bcr-Abl drugs in TOM-1 and BV-173 cell lines (**A**) MTS assays of TOM-1 cells treated with ZSTK474, BYL719, IPI145, Imatinib, Nilotinib and GZD824 alone or with the combinations indicated in the graph legends. The analysis was performed after 48 h. Results are the mean of three separate experiments. SD was less than 10%. (**B**) MTS assays of BV-173 cells treated with ZSTK474, BYL719, IPI145, Imatinib, Nilotinib and GZD824 alone or with the combinations indicated in the graph legends. The analysis was performed after 48 h of treatment. Results are the mean of three separate experiments. SD was less than 7%. ZSTK474, BYL719, IPI145, Imatinib, Nilotinib and GZD824 inhibitors were abbreviated in ZSTK, BYL, IPI, IMA, NIL and GZD, respectively.

As representative of the combined treatments, MTS assays on TOM-1 and BV-173 cell lines were also performed with ZSTK474/Imatinib,BYL719/Nilotinibor IPI145/GZD824. As expected, the combination of the drugs resulted in a potent synergism also at low concentrations of each inhibitor in these cell lines (Figure 7A and 7B).

### PI3K isoforms inhibition combined with anti Bcr-Abl drugs increased apoptotic effects in Ph^+^ B-ALL cells

**Figure 8:**
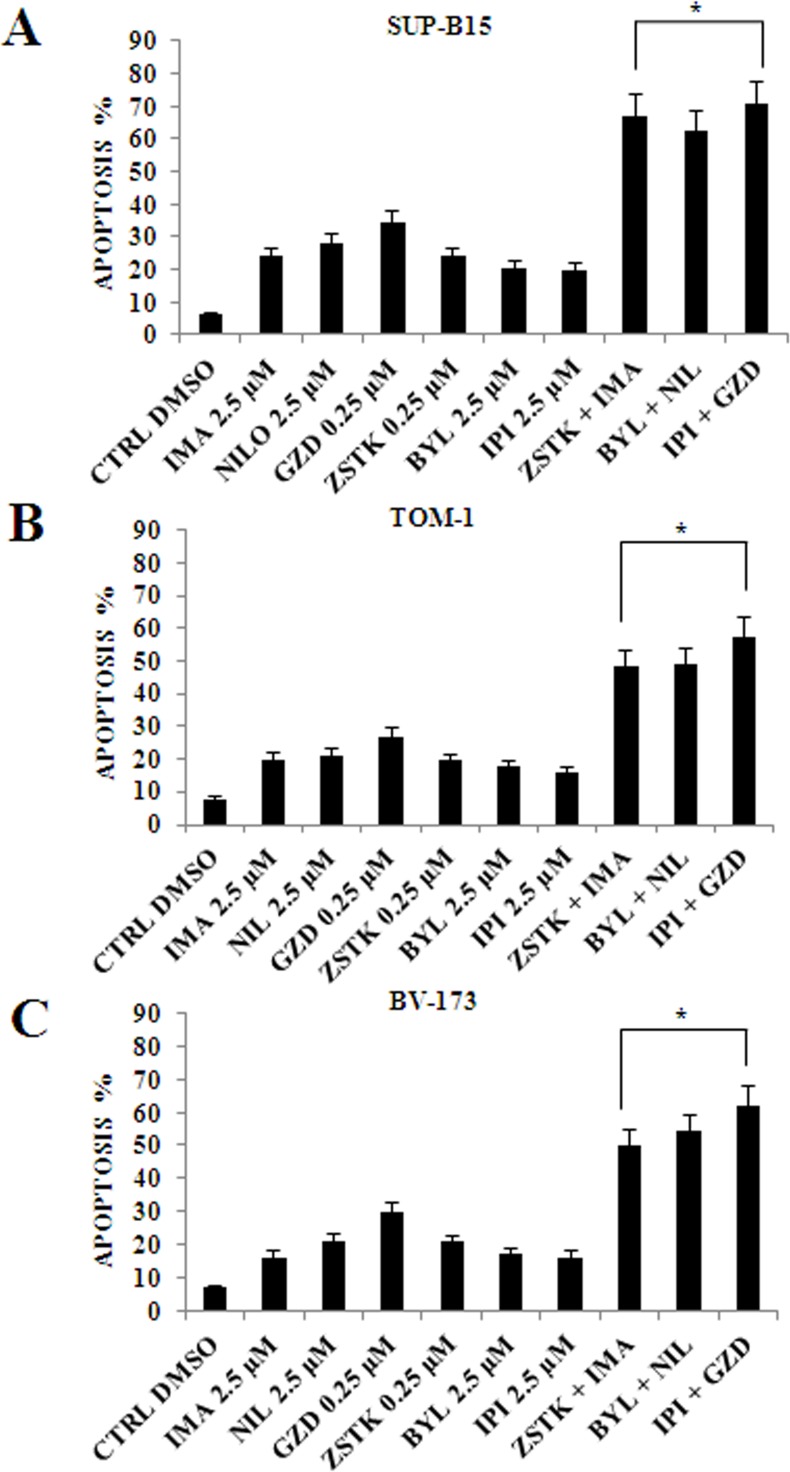
Flow cytometric analysis of drug-induced apoptosis by using selected PI3K isoform inhibitors in combination with anti Bcr-Abl drugs in Ph^+^ B-ALL cell lines (**A**) Analysis of Annexin-V positive cells after ZSTK474, BYL719, IPI145 and Imatinib, Nilotinib, GZD824 treatment alone or in combination in SUP-B15 cells. The analysis was performed after 24 h of treatment with 2.5 μM Imatinib, Nilotinib, BYL719 and IPI145 inhibitors, and 0.25 μM GZD824 and ZSTK474 drugs. The data are representative of three experiments ± SD. (**B**) Analysis of Annexin-V positive cells after ZSTK474, BYL719, IPI145 and Imatinib, Nilotinib, GZD824 treatment alone or in combination in TOM-1 cells. The analysis was performed after 24 h of treatment with 2.5 μM Imatinib, Nilotinib, BYL719 and IPI145 inhibitors, and 0.25 μM GZD824 and ZSTK474 drugs. The data are representative of three experiments ± SD. (**C**) Analysis of Annexin-V positive cells after ZSTK474, BYL719, IPI145 and Imatinib, Nilotinib, GZD824 treatment alone or in combination in BV-173 cells. The analysis was performed after 24 h of treatment with 2.5 μM Imatinib, Nilotinib, BYL719 and IPI145 inhibitors, and 0.25 μM GZD824 and ZSTK474 drugs. All samples were analyzed by the Muse^™^ Cell Analyzer. Results are the mean of three different experiments ± SD. In all figures asterisks indicate significant differences compared to control and single treatments (*p < 0.05). In all figures Control (untreated cells), ZSTK474, BYL719, IPI145, Imatinib, Nilotinib and GZD824 inhibitors were abbreviated in CTRL, ZSTK, BYL, IPI, IMA, NIL and GZD, respectively.

The apoptotic effects of the single and combined drug administration compared to the control in the three cell lines was then evaluated (Figure 8A–8C). The analysis was performed by Annexin-V-FITC staining and PI in flow cytometry after 24 h of drug treatments. The drug concentrations used were 0.25 μM for ZSTK474 and GZD824, and 2.5 μM for the other inhibitors.

The combined administration of the PI3K inhibitors with anti Bcr-Abl drugs showed a significant increase in the percentage of apoptotic cells.

### PI3K isoforms inhibition combined with anti Bcr-Abl drugs induced increased autophagy in Ph^+^ B-ALL cells

**Figure 9:**
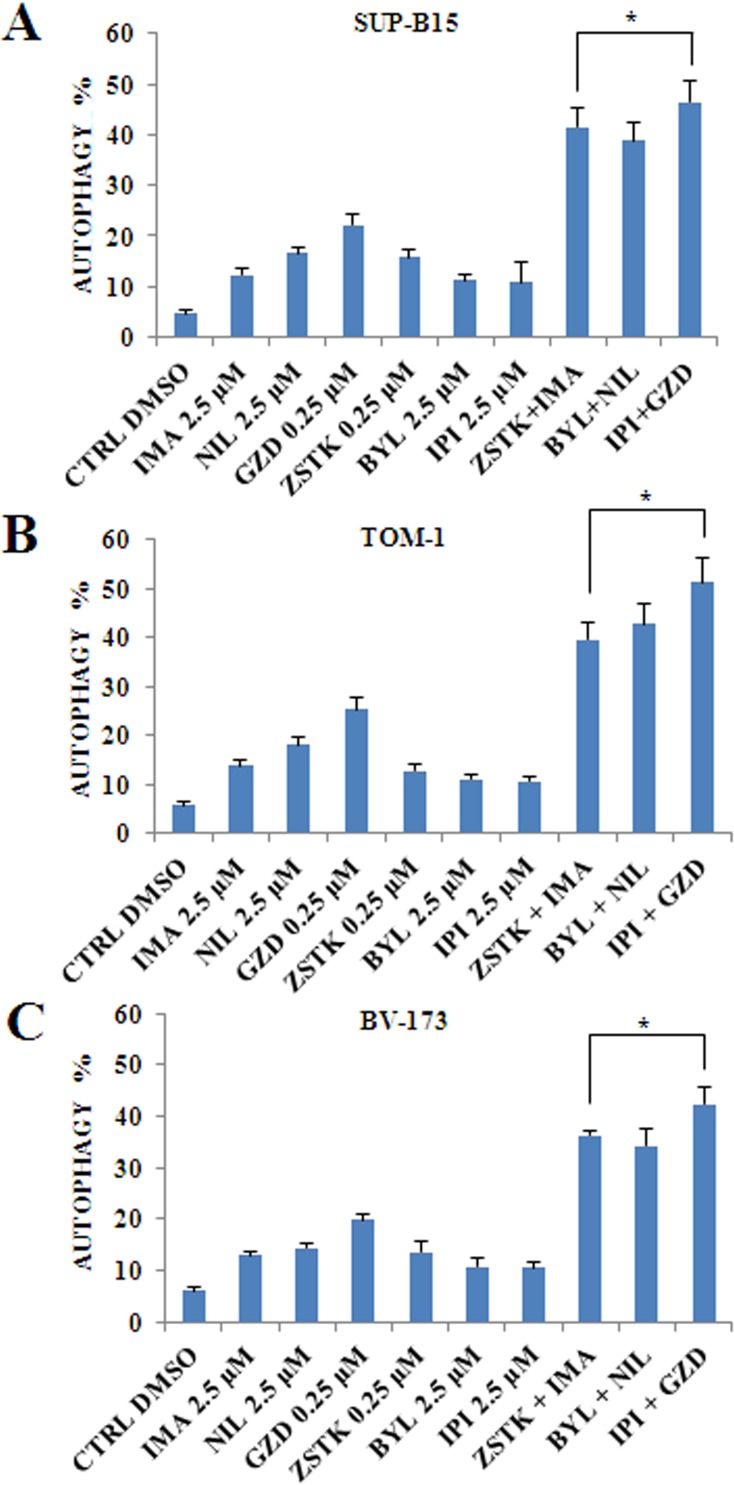
Flow cytometric analysis of drug-induced autophagy by using selected PI3K isoform inhibitors in combination with anti Bcr-Abl drugs in Ph^+^ B-ALL cell lines (**A**) Flow cytometric analysis of autophagy LC3 marker in SUP-B15 cell line treated with single drugs or combined administration of Imatinib, Nilotinib, GZD824 with ZSTK474, BYL719 and IPI145. The analysis was performed after 24 h of treatment with 2.5 μM Imatinib, Nilotinib, BYL719 and IPI145 inhibitors, and 0.25 μM GZD824 and ZSTK474 drugs. (**B**) Flow cytometric analysis of autophagy LC3 marker in TOM-1 cell line treated with single drugs or combined administration of Imatinib, Nilotinib, GZD824 with ZSTK474, BYL719 and IPI145. The analysis was performed after 24 h of treatment with 2.5 μM Imatinib, Nilotinib, BYL719 and IPI145 inhibitors, and 0.25 μM GZD824 and ZSTK474 drugs. (**C**) Flow cytometric analysis of autophagy LC3 antibody in BV-173 cell line treated with single and combined administration of Imatinib, Nilotinib, GZD824 with ZSTK474, BYL719 and IPI145. The analysis was performed after 24 h of treatment with 2.5 μM Imatinib, Nilotinib, BYL719 and IPI145 inhibitors, and 0.25 μM GZD824 and ZSTK474 drugs. All samples were analyzed by the Muse^TM^ Cell Analyzer. In A-C results are the mean of three different experiments ± SD. In all figures asterisks indicate significant differences compared to control and single treatments (*p < 0.05). In A–C control (untreated cells), ZSTK474, BYL719, IPI145, Imatinib, Nilotinib and GZD824 inhibitors were abbreviated in CTRL, ZSTK, BYL, IPI, IMA, NIL and GZD, respectively.

To better quantify autophagy, the detection of LC3A/B was performed in all cell lines by flow cytometry. After a 24 h incubation, a statistically relevant increase in autophagy was induced by combined treatment of TKIs and PI3K inhibitors when compared to single administrations (Figure 9A–9C).

## DISCUSSION

It has been reported that targeted cancer therapy could be more effective and less harmful than conventional chemotherapy [44]. PI3K plays a key role in regulating cell cycle, survival and metabolism, and the PI3K signaling cascade is a very frequently altered axis in human tumors [45]. Different compounds targeting members of the PI3K have been synthesized and are now in clinical trials [36]. Better understanding of the PI3K pathway modulation have led to a rational development and utilization of PI3K inhibitors in patients with leukemias. The pan-PI3K ZSTK474 inhibitor and the dual γ/δ IPI145 inhibitor are undergoing analysis and are already demonstrating increasing preclinical and clinical results [23, 46]. For example, ZSTK474 inhibitor displayed a potent anticancer activity in human tumor xenografts [47] while BYL719 has shown synergistic antineoplastic efficacy when used in endocrine cure against ER^+^/PIK3CA mutated breast tumor cells [48].

In Ph^+^ B-ALL cells, relapses are very frequent, especially in adults, with a very poor prognosis, highlighting the need for new therapeutic strategies [49]. Moreover, PI3K network has been strongly involved either in malignant transformation and in the development of TKI-resistance in Ph^+^ B-ALL [50].

We explored the therapeutic potential of the PI3K pan-inhibitor ZSTK474, the p110α inhibitor BYL719 and dual γ/δ inhibitor IPI145 in a set of Ph^+^ B-ALL cells. It is worth noting that these cell lines showed an hyperphosphorylation of PTEN, that results in its inactivation and hyperactivation of PI3K/Akt/mTOR signaling network [51].

We reported that ZSTK474 reduced cell viability and caused both autophagy and apoptosis in Ph^+^ B-ALL cells. ZSTK474, when administered in the micromolar range, synergized in all cell lines with either Imatinib, Nilotinib or GZD824. In this study, we analyzed for the first time the efficacy of PI3K isoform-selective inhibitors in Ph^+^ B-ALL. BYL719 and IPI145 were cytotoxic in the micromolar range to Ph^+^ B-ALL cell lines, as shown by MTS and Annexin V-stained samples analysis. However, ZSTK474 was more cytotoxic than BYL719 and IPI145, as it displayed an IC_50_ value of 0.5 μM. The phosphorylation level of the key components of the PI3K/Akt/mTOR axis, analyzed by Western blot, showed the same sensitivity to ZSTK474 inhibition in all cell lines. It should be highlighted that another recent study has demonstrated the efficacy of the pan-PI3K inhibitor, NVP-BKM120, in pre-clinical models of Ph^+^ B-ALL [52]. Interestingly, this inhibitor displayed an IC_50_ of 0.5 μM which is the same as that of ZSTK474.

However, it should be considered that NVP-BKM120 displays considerable off-target effects [53] which so far have not been reported for ZSTK474.

It is also important to emphasize that a previous study from our group has documented how ZSTK474 was not cytotoxic to healthy T-cells [54].

A greater efficacy was also evident in case of GZD824, a third generation anti Bcr-Abl drug, which showed an increased anti-leukemic activity in comparison to first and second generation inhibitors in Ph^+^ B-ALL cells.

When cell lines were treated with anti Bcr-Abl drugs the phosphorylated form of CrkL protein was abolished, whereas the phosphorylated S6 ribosomal protein was minimally affected. Interestingly, these findings are in agreement with those of others who documented in their study that Akt activity was surprisingly not inhibited by Imatinib administration of primary CML-cells [55].

This demonstrated the specificity of these inhibitors against the Bcr-Abl fusion protein. In contrast, cell samples treated with selected PI3K isoform inhibitors showed an unchanged expression level of phosphorylated CrkL protein and a strong decrease in the phosphorylated S6 ribosomal protein.

Overall, the PI3K isoform inhibitors used in this study displayed increased potency when combined with anti Bcr-Abl drugs in terms of cell viability reduction as well as, apoptosis and autophagy induction. Future studies should address the issue of how these drug combinations increase apoptosis and autophagy.

In conclusion, our data show that investigational PI3K inhibitors can block the growth of Ph^+^ B-ALL cell lines but are likely to be most effective when used in combination with Bcr-Abl inhibitors and provide an important preclinical rationale for future clinical applications.

## MATERIALS AND METHODS

### Materials

RPMI-1640 and McCoy's 5A medium, fetal bovine serum (FBS), penicillin and streptomycin were purchased from Lonza Milano SRL (Milan, Italy). ZSTK474, BYL719, TGX221, AS605240, CAL101, IPI145, Imatinib, Nilotinib and GZD824 were obtained from Selleck Chemicals (Houston, TX, USA). For cell viability determination, CellTiter 96 Aqueous One Solution Cell Proliferation Assay (MTS) was purchased from Promega (Milan, Italy). Annexin V/7-ADD detection kit was from Merck-Millipore (Darmstadt, Germany). Western blot antibodies for total Akt-1, Ser473 p-Akt-1 and Thr308 p-Akt-1 were from Santa Cruz Biotechnology (Santa Cruz, CA, USA), while all the other antibodies were from Cell Signaling Technology (Danvers, MA, USA), including the rabbit secondary antibody. Chloroquine, the mouse secondary antibody and the monoclonal β-Actin antibody were purchased from Sigma Aldrich (Milan, Italy). Signals were detected using ECL Plus reagent from Perkin Elmer (Boston, MA, USA).

### Cell cultures

The SUP-B15 Ph^+^ B-ALL cell line was obtained from Deutsche Sammlung von Mikroorganismen und Zellkulturen GmbH (Braunschweig, Germany). BV-173 and TOM-1 Ph^+^ B acute lymphoblastic leukemia (B-ALL) cell lines were obtained from Dr. Fabrizio Pane's laboratory at University of Naples, Italy. BV-173 and TOM-1 were maintained in RPMI-1640 medium supplemented with 20% heat-inactivated fetal bovine serum (FBS), 100 units/ml penicillin and 100 mg/ml streptomycin at a density of 0.5 to 2 × 10^6^ cells/ml and were incubated at 37°C with 5% CO_2_.

SUP-B15 was maintained in McCoy's 5A medium supplemented with 20% heat-inactivated fetal bovine serum (FBS), 100 units/ml penicillin and 100 mg/ml streptomycin at a density of 0.5 to 2 × 10^6^ cells/ml and were incubated at 37°C with 5% CO_2_.

### Western blot

The cells were homogenized for 30 min in cold lysis buffer (50 mM Hepes pH 7.5, 5 mM EDTA pH 8.0, 10 mM MgCl2, 150 mM NaCl, 50 mM NaF, 20 mM β-glicerophosphate, 0.5% NP40, 0.1 mM sodium orthovanadate, 1 mM PMSF, 1 mM DTT and protease inhibitor cocktail, Roche Applied Science Basel, Switzerland). Lysate was purified by centrifugation for 10 min at 4°C and 20–50 μg of solubilized proteins were resolved on 10% or 12% SDS-PAGE [56].

### Cell viability analysis

MTS (3-[4,5-Dimethylthythiazol-2-yl]-5-(3-carboxy methoxyphenyl)-2-(4-sulfophenyl)-2H-tetrazolium, inner salt assay was performed to assess the sensitivity of cells to drugs, as previously described [57].

### PI/Annexin V assay

Apoptosis analysis was performed by staining with Annexin V/7-AAD, using the Muse^™^ Cell Analyzer (Merck Millipore, Milan, Italy) in according to the manufacturer's instructions. In brief, a 100 μl treated cell suspension was labeled for 20 min in the dark with the same volume of the Muse^TM^ Annexin-V & Dead Cell reagent (Merck Millipore). Subsequently, quantitative detection of Annexin-V/7-AAD positive cells was performed using the Muse^™^ Cell Analyzer.

### Autophagy analysis and detection of endogenous LC3

Autophagy analysis was performed using the Muse^™^ Cell Analyzer (Merck Millipore, Milan, Italy). In brief, 8 × 10^4^ cells were plated in 96 well plates and treated with the different drugs for 24 h. Then, cells were harvested, treated with Autophagy Reagent A for 2–6 h, washed with Assay Buffer, incubated for 30 minutes in the dark with Anti-LC3 Alexa Fluor^®^555 Antibody and acquired by Muse. Samples were then analyzed according to the instrument protocol.

### Caspase 3/7 activity assay

Caspase activity was measured with the Apo-One Homogeneous Caspase 3/7 assay kit (Promega Corporation, Madison, WI, USA), according to the manufacturer's instructions. The induction of apoptosis and associated activation of caspases 3 and 7 were measured by enzymatic cleavage of the profluorescent substrate rhodamine 110, bis-N-CBZ-L-aspartyl-Lglutaml- L-valyl-L-aspartic acid amide (Z-DEVD-R110), which releases the intensely fluorescent rhodamine 110-cleaving group. Cells were seeded at a density of 1 × 105/ml and incubated in a 96-well plate in the presence or absence of drug for 48 h. 100 μl of the homogeneous caspase-3/-7 reagent was added to each well and the reaction mixture was incubated for 2 h at room temperature. Fluorescence was measured at an excitation wavelength of 485 nm and an emission wavelength of 538 nm. Results are expressed as relative fluorescence units (RFU), as previously described [54].

### Combined drug effect analysis

The effects and the potential synergy of drug combinations were evaluated from quantitative analysis of dose-effect relationship, as described previously [58].

For each experiment, a combination index (CI) number was calculated using the Biosoft CalcuSyn software (Biosoft, Cambridge, UK). This method of analysis generally defines CI values from 0.9 to 1.1 as additive, from 0.3 to 0.9 as synergistic and below 0.3 as strongly synergistic, whereas values over 1.1 are considered as antagonistic.

### Statistical evaluation

The data are presented as mean values from three separate experiments ± SD. Data were statistically analyzed by a Dunnet test after one-way analysis of variance (ANOVA) at a level of significance of *P* < 0.05 vs control samples [59].

## Abbreviations

A: AS605240
Abl: Abelson
Akt: Protein kinase B
AS: AS605240
B-ALL: B-Acute Lymphoblastic Leukemia
Bcr: Breakpoint cluster region
BYL: BYL719
C: CAL101
CAL: CAL101
CHL: Chloroquine
CI: Combination Index
CML: Chronic Myeloid Leukemia
CTRL: Control
GZD: GZD824
IMA: Imatinib
IPI: IPI145
mTOR: Mammalian Target of Rapamycin
NIL: Nilotinib
PARP: Poly (ADP-ribose) polymerase
Ph^+^: Philadelphia chromosome positive
PI: Propidium Iodide
PI3K: phosphoinositide 3-kinase
PI3Ks: Class I phosphatidylinositol 3-kinases
PTEN: Phosphatase and tensin homolog
S6RP: S6 Ribosomal Protein
TGX: TGX221
TKI: Tyrosine Kinase Inhibitor
ZSTK: ZSTK474
